# Long-term follow-up after surgical repair of abdominal rectus diastasis: A Prospective Randomized Study

**DOI:** 10.1177/1457496920913677

**Published:** 2020-04-17

**Authors:** Ebba Swedenhammar, Karin Strigård, Peter Emanuelsson, Ulf Gunnarsson, Birgit stark

**Affiliations:** Department of Molecular Medicine and Surgery, Karolinska Institutet, Karolinska University Hospital, Solna (L1:00), Stockholm, SE-171 76, Sweden; Division of Surgery, Department of Surgical and Perioperative Sciences, Umeå University, Karolinska University Hospital, Umeå, Sweden; Department of Molecular Medicine and Surgery, Karolinska Institutet, Stockholm, Sweden; Division of Surgery, Department of Surgical and Perioperative Sciences, Umeå University, Karolinska University Hospital, Umeå, Sweden; Department of Molecular Medicine and Surgery, Karolinska Institutet, Stockholm, Sweden

**Keywords:** Abdominal rectus diastasis, operation method, abdominoplasty, quality of life, recurrence, long-term follow-up

## Abstract

**Background:**

Abdominal rectus diastasis can lead to functional disability. There is no consensus regarding treatment. This was a prospective study on patients randomized to surgery using either Quill self-retaining sutures or retromuscular mesh for abdominal rectus diastasis repair. The primary aim of the study was to compare long-term recurrence after surgery. Secondary aims were abdominal muscle strength, pain, and quality of life.

**Methods:**

A total of 57 patients were eligible and 52 were investigated. A routine 1-year follow-up ruled out any patient with recurrence and this was followed up by clinical examination for recurrence and assessment of the secondary outcomes a median of 5 years (3.8–6.5 years) after surgery. Quality of life was assessed using the Short Form-36 questionnaire. Pain related to activity was evaluated using the Ventral Hernia Pain Questionnaire.

**Results:**

No recurrence of abdominal rectus diastasis was found. Significant improvements were seen between index surgery and long-term follow-up in all domains of Short Form-36. There were no significant differences in quality of life or self-reported muscle strength between the two surgical groups. Long-term pain remained unchanged compared to that at the 1-year follow-up. “Pain this week” had decreased significantly at long-term follow-up compared to prior to surgery (mesh p = 0.009, Quill p = 0.003).

**Conclusions:**

No recurrence of abdominal rectus diastasis appeared. There was no difference in quality of life or long-term pain between the two surgical groups. Implantation of retromuscular mesh entails more extensive surgery implying potentially higher risk for complications. This leads us to recommend reconstruction with double-row self-retaining sutures for the repair of abdominal rectus diastasis in patients with functional disability.

## Introduction

Abdominal rectus diastasis (ARD) can be either a primary or a secondary condition following pregnancy, massive weight loss, or previous abdominal surgery. ARD affects both genders but is more frequent in women, often related to hormonal variations in pregnancy^1–3^. The incidence of ARD after delivery is not known with certainty, with studies reporting a wide range between 40% and 60% after 4 days to 1 year^4^. ARD is present when the linea alba width is more than 27 mm at umbilical level, or approximately more than 1 cm above or below the umbilicus depending on age^2^. Midline bulging of the anterior abdominal wall can be perceived as discomfort, pain, or impaired core stability. Difficulty in performing daily activities or during physical activity has been reported^5,6^.

No consensus has been reached regarding the most appropriate surgical method or associated benefits. Different techniques for ARD repair have been described in several small studies and they differ in respect to the number of layers of sutures, suture material used, positioning of the sutures, and if a mesh was used^7,8^. A prospective randomized study by our group^9^ compared surgical repair with plication using double-row Quill sutures, to retromuscular mesh repair. A group allocated to dedicated exercises served as controls at a 3-month follow-up. By the 1-year follow-up, the operated patients had increased abdominal muscular strength measured by the Biodex dynamometer, experienced less pain during daily activities, and reported improved quality of life (QoL)^10^. Moreover, the double-row repair with Quill sutures did not result in more postoperative complications or recurrence of ARD compared to the mesh repair^9^. The consistency of these findings needed to be addressed in a long-term follow-up analysis.

The aim of this study was to compare long-term outcomes of ARD repair using double-row Quill self-retaining suture with retromuscular mesh repair in patients with functional disability due to ARD. The primary endpoint was recurrence rate of diastasis after the 1-year follow-up. Secondary endpoints were QoL, self-reported abdominal muscle strength, and pain in the abdominal wall.

## Materials and Methods

### Study Design and Participants

Patients with the diagnosis ARD combined with discomfort and/or abdominal pain, referred to either the Department of Reconstructive Plastic Surgery or the Centre for Surgical Gastroenterology at the Karolinska University Hospital between December 2009 and December 2012 were invited to take part in the study. Inclusion and exclusion criteria for the primary study by Emanuelsson et al.^9^ are shown in Table 1. Patients underwent computed tomography (CT) and were measured clinically prior to the randomization. The patients were referred to either site depending on the patients’ proximity to the hospital sites. Eligible patients were randomized prior to the operative procedure to either one of two surgical procedures: double-row vertical suture repair with self-retaining barbed sutures 2/0 PDO (Quill™SRS)^11^ or reinforcement with lightweight polypropylene mesh (BARD™ Soft Mesh) placed in the retromuscular plane on the peritoneum, the mesh was not anchored laterally with sutures. Then the anterior fascia was closed with running sutures 2/0 PDS (polydioxanone). For technical reasons, a wide dissection from the pubic symphysis to the xiphoid was done to expose the rectus muscles completely. Further details of the operative procedures have been described elsewhere^9^. A full abdominoplasty was performed if the patient had surplus skin.

**Table 1.** Inclusion and exclusion criteria from the primary study as seen in co-author Emanuelsson et al.’s^9^ article.

Inclusion	Exclusion
ARD ⩾ 3 cm	Ongoing pregnancy
>18 years old	Ongoing breastfeeding
Abdominal wall discomfort or tenderness	Immunosuppressive therapy
Wish to have abdominal wall reconstruction	Smoking
For women: ⩾1 pregnancy, >1 year after childbirth	

ARD: abdominal rectus diastasis.

**Fig. 1.**
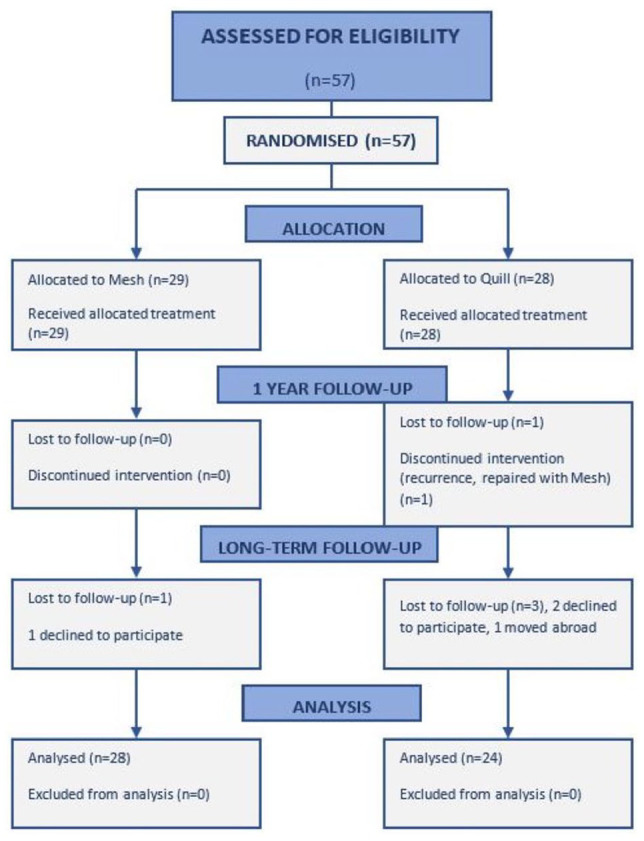
Flow chart: consort diagram. Patients eligible for intervention and follow-up.

A consort diagram for the study is shown in Fig. 1. For the calculated power (80%) in the primary study, 25 patients were needed in each arm. For every drop-out, another three patients were included to maintain this power. At the primary randomization, 57 patients were randomized to surgery and 32 to training. The surgical randomization took place when the patient had been anesthetized in the operating theater. All surgeries were executed by a colorectal surgeon with special interest in abdominal wall surgery and a plastic surgeon in collaboration. Patients were evaluated between 26 October 2015 and 28 September 2016. The median and mean long-term follow-up was 5 years (range: 3.8–6.5 years, interquartile range (IQR): 1.2).

The study was approved by the Regional Ethics Review Board in Stockholm (D.nr 2009/227-31, 2011/1186-32, 2016/55-32) and was registered on ClinicalTrial.gov with the number 2009/227-31/3/PE/96. The Declaration of Helsinki principles of ethical standard were followed.

### Self-Reported Questionnaires

Aspects of QoL as well as pain and its effect on daily activities were addressed using the Short Form-36 (SF-36) questionnaire and the Ventral Hernia Pain Questionnaire (VHPQ)^12^. SF-36 is an instrument designed to create health scores in eight different dimensions. There are four mental and four physical dimensions that can also be summarized in two component scores^13^. The VHPQ is a validated questionnaire to assess the patient’s own experience of pain before and after surgery of the ventral abdominal wall and relates to daily activities. Outcomes were compared with preoperative and 1-year follow-up data.

### Clinical Investigation

At the long-term follow-up, all patients underwent clinical assessment by a senior surgeon not previously involved in any part of the study.

Recurrence of ARD was defined as separation of the rectus abdominis muscles ⩾3 cm, either above or below the umbilicus. Measurements of ARD were made using exactly the same method as in the examination prior to surgical repair^9^. Any gap present was measured halfway between the pubic symphysis and the umbilicus or halfway between the xiphoid process and the umbilicus. If recurrence was uncertain in the clinical assessment, CT was performed.

The abdominal circumference was measured at the level of the umbilicus. Potential soft-tissue irregularities covering the abdominal wall, as well as appearance of the scar and position of the umbilicus were noted. Details of medical events, smoking, or further pregnancies since the 1-year follow-up were retrieved from patient notes or taken at the long-term follow-up visit.

### Statistics

Statistica version 13 was used for all statistical calculations. Patient demographics were presented with min–max and IQR. When comparing continuous variables, the Mann–Whitney U test was used since non-parametric outcomes were expected. Dichotomous data were compared with the chi-square test and Fischer’s exact test. The SF-36 results were evaluated with paired and independent *t* tests. Collected data were matched with reference data from an age-matched Swedish population^13^. The VHPQ was evaluated using the Mann–Whitney U test and dependent variables with Wilcoxon’s rank-sum test.

Power was originally calculated for the primary endpoint in the original study, recurrence at the 1-year follow-up, in our previous study on ARD repair. To obtain a significance level of 95% for 80% power, each surgical group required at least 25 patients^10^ assuming a recurrence rate of 30% in the Quill group and 5% in the mesh group after 1 year. This presumption was based on the results of previous studies on incisional hernia repair with mesh or sutures^14, 15^.

## Results

Of the 57 patients operated, 53 were available for long-term follow-up (Fig. 1 and Table 2). One early recurrence in the Quill group was repaired with retromuscular mesh within 6 weeks after index surgery. This patient was excluded from further follow-up within the frame of our research protocol. There were no significant differences in demographic parameters between the groups (Table 2). Except for the early recurrence before the 3-month follow-up, no ARD recurrence was found in either group between the 1-year and long-term follow-up. Two patients (one in each group) noted some bulging of the abdominal wall and some diffuse abdominal pain, but no recurrence was confirmed either by clinical assessment or by CT. Five patients had resumed smoking after the last follow-up.

**Table 2.** Demographics at long-term follow-up.

	Mesh	Quill	p
Follow-up since operation (years)			0.797
Median	4.95	5.10	
Min–max	4–6.5	3.8–6.3	
IQR	1.05	1.3	
BMI			0.700
Median	22.9	22.8	
Min–max	18.1–30.2	18.8–36	
IQR	3.85	4.95	
Age			
Median	43	42	0.776
Min–max	29–63	30–62	
IQR	6.5	11	
Gender, n (%)			1
Female	27 (96.4)	23 (95.8)	
Male	1 (3.6)	1 (4.2)	
Smokers, n (%)	1 (3.6)	4 (16.7)	0.169
Postoperative pregnancy, n (%)	2 (6.7)	2 (8.3)	1

BMI: body mass index; IQR: interquartile range.

Significant levels are calculated with Mann–Whitney U test and for dichotomous variables with chi-square test and Fischer’s exact test. The five smokers found in long time follow-up began smoking after surgery.

## Abdominal Wall Pain

VHPQ questionnaire ratings are listed in Table 3. No significant differences were seen between the two groups. When comparing dependent data, “pain this week” was significantly lower in both groups compared to preoperative values (preoperative vs long-term: mesh p = 0.009, Quill p = 0.003). There was not enough material for statistical analysis in several of the variables due to few symptoms at the long-term follow-up. At the long-term follow-up, a few patients mentioned the appearance of discomfort and diffuse pain that was not revealed in the two questionnaires.

**Table 3.** The VHPQ results for preoperative and long-term follow-up after repair.

Questionnaire	Preoperative	Long term	Preoperative	Long term
	Mesh (n = 29)	Mesh (n = 28)	Quill (n = 28)	Quill (n = 24)
Pain right now ⩽1	22	25	21	23
Pain right now >1	6	3	7	1
Pain last week >1	11	3	12	1
Difficulty rising from chair	2	0	7	0
Difficulty sitting	1	1	3	2
Difficulty standing	1	1	6	1
Difficulty climbing stairs	2	0	6	0
Difficulty driving a car	1	0	0	0
Difficulty performing sports and physical activity	11	5	14	3

VHPQ: Ventral Hernia Pain Questionnaire.

If patients graded their pain right now as ⩽1, the pain was considered easily ignored. Scorings higher than 1 constituted pain not easily ignored during everyday activities. They presented with symptoms, for example, swelling after eating or discomfort and weakness in the abdominal trunk. Other reported symptoms were tactile discomfort, muscle cramps during exercise, less stamina during physical exercise, and lower back pain.

“Pain last week” was significantly lower in both groups compared to preoperative values (preoperative vs long-term: mesh p = 0.009, Quill p = 0.003).

## Core Stability and Overall Well-Being

Twenty-five patients (89.3%) in the mesh group and 21 patients (87.5%) in the Quill group expressed no difference in well-being compared to the improvement reached at the 1-year follow-up^10^. A similar situation was the case for self-reported core stability. Twenty-seven patients in the mesh group and 20 in the Quill group were satisfied with functional outcome, but only 11 versus 7 patients, respectively, were satisfied with the aesthetic outcome. Excess skin at the lateral borders of the lower abdominal scar, irregularities of the fat layer covering the abdominal wall, and a wider scar than expected were the main complaints.

### SF-36

**Fig. 2.**
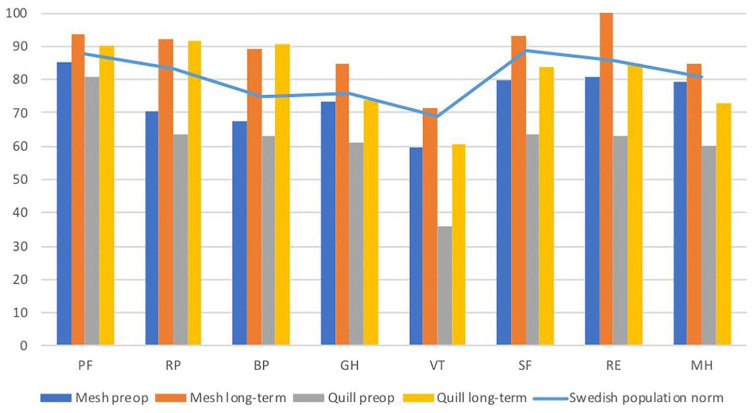
SF-36. Results from the two groups, before operation and at long-term follow-up after surgery, compared to a matched Swedish population. PF: physical functioning; RP: role-physical; BP: bodily pain; GH: general health; VT: vitality; SF: social functioning; RE: role-emotional; MH: mental health.

Prior to surgery, both groups scored significantly lower in all domains compared to the Swedish matched population (p < 0.001)^9^. Furthermore, the baseline preoperative physical and mental health scores of patients in the Quill group were significantly lower than those reported in the mesh group. All domains were above the Swedish matched population at the time of the long-term follow-up, except for vitality (VT), social function (SF), and mental health (MH) in the Quill group (Fig. 2).

When comparing mental component score (MCS) between the two groups, a significantly higher score was seen in the mesh group (p = 0.002). There was no difference in physical component score (PCS) between the groups (p = 0.867).

### Pregnancy

Two patients in each group became pregnant during the period between the 1-year and long-term follow-up. Interestingly, the two patients operated with Quill suture plication described recurrence of ARD during the second respective third trimester with return to stability of the abdominal wall after delivery. The two patients operated with retromuscular mesh suffered from abdominal wall pain and discomfort in the second respective third trimester. They experienced intense rigidity of the anterior abdominal muscles with more lateral than midline expansion of abdominal wall tissues.

### Clinical Outcome

The circumference of the waist was similar in both groups. No significant difference was seen between the two groups when comparing body mass index (BMI) and circumference prior to surgery with values at the long-term follow-up (Table 4).

**Table 4.** BMI and waist circumference preoperative measurements compared to long-term follow-up.

	BMI preoperative	BMI long term	p	Circumference preoperative	Circumference long term	p
Mesh			0.633			0.412
Median (min–max)	23 (18–30)	22.9 (18.1–30.2)		85.5 (71–102)	85 (71–102)	
IQR	4	3.85		12	8	
Quill			0.605			0.167
Median (min–max)	23 (18–31)	22.8 (18.8–36)		87 (72–116)	87.5 (60–113)	
IQR	4	4.95		19	17.5	

BMI: body mass index; IQR: interquartile range.

## Discussion

In a previous study of the same study cohort^10^, we showed that no recurrence of ARD had occurred at 1-year follow-up after the index operation. In the present long-term follow-up, no further recurrences occurred indicating that reconstructions of ARD with either mesh or Quill double-row suture are stable over time. Consequently, our hypothesis that there would be a difference in ARD recurrence between Quill double-row suture and retromuscular mesh repair (30% and 5%, respectively) 1 year after surgery was rejected, as well as throughout the long-term follow-up period in this prospective randomized trial. Thus, no method was overtly superior for ARD repair in this respect.

The present data are contrary to previous reports stating ARD recurrence rates between 30% and 40% for suture repair. Van Uchelen et al.^16^ reported a 40% recurrence rate after repair with a single-row vertical plication using absorbable sutures. In contrast to Gama et al.^17^, we did not see a 30% recurrence rate when using barbed sutures. This discrepancy in results could be explained by the double-row longitudinal suture technique used in this study, reducing horizontal tension at the medial margins of the rectus muscles. These results are also comparable to those from Nahas et al.^18^ and Rosen et al.^19^ using a similar approach. The risk for fascial rupture may thus be reduced compared to using a single-row technique^17^. Further randomized studies evaluating different suturing techniques are needed.

Our own observations had previously indicated a significant improvement in all domains and parameters 1 year after ARD repair^10^. In this assessment, the majority of patients experienced overall improved QoL and diminished bodily pain in the SF-36 and VHPQ. The consistency of results in the various SF-36 domains and VHPQ indicates that the outcome of surgery is long-lasting and stable over time.

At long-term follow-up, all domain scores were above the Swedish matched population, except for VT, SF, and MH in the Quill group. Preoperatively patients in the Quill group had a lower component score for self-rated mental health than patients in the mesh group. Furthermore, even though the demographics of the patient groups appeared to be similar, we noted that patients in the Quill group scored significantly lower for specific SF-36 domains. Meningaud et al. showed in their multicentre study that patients undergoing plastic surgery might have a different psychological profile compared to the general population. Using structured interviews and three assessment scales, they found more depression and anxiety among plastic surgery patients^20^. Nonetheless, the psychological profile of our two groups differed somewhat after randomization as shown in the preoperative SF-36 results.

The majority of patients in both groups were satisfied with their functional outcome (return from sick-leave, running marathons, and possibility to play with their children among others) but expressed dissatisfaction regarding the aesthetic outcome. This emphasizes the importance of addressing the patient’s expectations; aspects of functionality and aesthetics should be clearly explained at the preoperative visit. Further studies are needed to identify the patient cohort most likely to benefit from ARD repair. In-depth interviews in combination with assessment of abdominal function and QoL could possibly help to establish a score system for rating indication for surgery.

In the clinical examination, we found a median waist circumference that was larger than expected, in some cases the circumference was larger than prior to index surgery despite no ARD recurrence or weight gain. Up to our best knowledge verified by search in PubMed and Web of Science, this observation has not been described previously. Many patients had a low-to-normal BMI, but their waist circumference was larger than expected, the median in the mesh group was 85 cm compared to 87.5 cm in the Quill group. According to the World Health Organization (WHO), women with a waist circumference larger than 88 cm run a great risk for cardiovascular disease and an increased risk if the waist is 80–88 cm^21^. One could argue that these patients might have had a general laxity in the abdominal wall even though the repair was intact. Could this laxity be an expression of a difference in the muscular biology and morphology of the abdominal wall? This aspect will be addressed in further morphological studies of the muscles and connective tissue of the abdominal wall.

Four patients became pregnant after the 1-year follow-up at which time a few patients asked about future pregnancy. Nahas published a case report of a woman becoming pregnant 2.5 years later after abdominoplasty including diastasis repair with plication. According to Nahas, despite no recurrence of the diastasis, the patient’s waist had returned to normal 15 months after delivery. Nahas^22^ suggested delaying pregnancy at least 12 months after surgery to assure formation of mature fibrotic tissue after repair.

There are few randomized prospective studies concerning repair of rectus diastasis and even fewer with long-term follow-up. The dropout rate in this study was low and we were able to collect a wide range of parameter values that could be compared with preoperative data. It would be valuable to identify specific markers and symptoms that correspond to ARD and changes over time after the index operation. There was an obvious risk for bias when comparing the two groups regarding self-reported mental health since patients in the Quill group scored poorer mental health even prior to surgery.

## Conclusion

No recurrence of ARD developed between the 1-year and a long-term follow-up after repair with double-row barbed sutures or retromuscular mesh in this prospective randomized study. Results of ARD repair were stable during long-term follow-up also regarding improvement in QoL. Included patients had an ARD width of 3–7 cm with a median of 4 cm. Implantation of retromuscular mesh entails more extensive surgery than double-row suture repair, thus having a higher potential risk for complications. This leads us to recommend using double-row self-retaining suture for the repair of ARD. More studies are needed, because for this group of patients an improvement in QoL and less pain can be of great importance.
